# Combination of violet light irradiation and collagenase treatments in a rabbit model of keratoconus

**DOI:** 10.3389/fmed.2023.1109689

**Published:** 2023-05-24

**Authors:** Hidenaga Kobashi, Takashi Yano, Kazuo Tsubota

**Affiliations:** ^1^Department of Ophthalmology, School of Medicine, Keio University, Tokyo, Japan; ^2^Tsubota Laboratory Inc., Tokyo, Japan; ^3^Life Science Laboratories, Ltd., Osaka, Japan

**Keywords:** keratoconus, animal model, collagenase, violet light, corneal cross-linking

## Abstract

**Purpose:**

We evaluated the use of collagenase treatment to generate a rabbit model of keratoconus and the impact of violet light (VL) irradiation on the disease model in six Japanese White rabbits.

**Methods:**

After epithelial debridement, the collagenase group was treated with a collagenase type II solution for 30 min; the control group was treated with a solution without collagenase. Three rabbits also underwent VL irradiation (375 nm, irradiance 310 μW/cm^2^) for 3 h daily for 7 days after topical collagenase application. Slit-lamp microscopy results, steep keratometry (Ks), corneal astigmatism, central corneal thickness, and axial length were examined before and after the procedure. The corneas were obtained on day 7 for biomechanical evaluation.

**Results:**

A significant increase in Ks and corneal astigmatism was observed in the collagenase and VL irradiation groups compared with the control group on day 7. No significant difference was found in the change in corneal thickness between the groups. The elastic modulus at 3, 5, and 10% strain was significantly lower in the collagenase group than in the control group. There was no significant difference in the elastic modulus at any level of strain between the collagenase and VL irradiation groups. The average axial length at day 7 was significantly longer in the collagenase and VL irradiation groups than in the control group. Collagenase treatment induced a model of keratoconus by steepening the keratometric and astigmatic values. There was no significant difference in the observed elastic behavior of normal and ectatic corneas under physiologically relevant stress levels.

**Conclusion:**

VL irradiation did not cause regression of corneal steepening in a collagenase-induced model during short-term observation.

## Introduction

Keratoconus (KC) is a progressive, frequently asymmetric, and corneal thinning disorder characterized by changes in the structure and organization of corneal collagen (1, 2). It results in corneal thinning and protrusion, progressive myopia, and irregular astigmatism. It is difficult to establish an *ex vivo* and *in vivo* corneal ectatic model of KC. Previous studies have examined the treatment of corneal ectasia using excimer lasers, but the corneal shape did not result in a KC model (3, 4). Hong et al. (5) reported a significant increase in corneal curvature in human donor corneas after topical collagenase application. Qiao et al. (6) mimicked KC with increased corneal keratometric parameters, corneal thinning, and a lower elastic modulus using rabbits. Wang et al. (7) developed an *ex vivo* corneal ectatic model that mimics KC by applying collagenase and chondroitinase enzymes to rabbit corneas. However, other parameters, such as corneal astigmatism and axial length, have not been investigated. Ihalainen et al. (8) reported increased collagenase and gelatinase activities in the medium of human KC corneal cultures. The lack of preclinical animal models of KC has been an obstacle for evaluating potential new therapies. Because abnormal collagenase activity and decreased total collagen levels are present in KC, exposure of collagenous enzymes to normal tissue may simulate some aspects of acute KC disease.

Recently, we reported a novel technology, KeraVio, consisting of violet light (VL) irradiation and riboflavin treatment, in rabbit and human corneas (9). KeraVio halted disease progression in eyes with corneal ectasia, similar to corneal cross-linking (CXL). KeraVio treatment uses an eyeglass with a 375-nm-wavelength VL source applied to the cornea, and the patients wear the eyeglass daily without limitations. Corneal epithelial peeling in CXL not only induces ocular pain after surgery but also carries a potential risk of corneal infection. Some modified CXL-associated accelerated and/or transepithelial techniques have recently become popular in the treatment of KC (10, 11). KeraVio treatment avoids these complications of CXL surgery and may become another option for KC treatment, but its efficacy has not been compared with that of CXL. To simplify the treatment procedure, we preliminarily identified originally endogenous riboflavin in the human cornea without adding riboflavin drops, and a relatively low intensity of VL irradiation itself strengthened the corneal stiffness in porcine corneas (12). In a normal eye, VL is absorbed by the cornea, which contains physiological riboflavin and other photosensitizers, leading to CXL in the cornea. If physiological riboflavin originally exists in the human cornea, VL irradiation may strengthen the corneal stiffness without adding riboflavin drops in the KeraVio technique. Subsequently, a clinical trial of KeraVio without riboflavin drops was launched (jRCTs032190267). To optimize the KeraVio treatment protocols based on the dose of riboflavin and VL irradiation, we need to develop an experimental model of KC. In this study, KeraVio was defined as a treatment using VL to stop the progression of KC, regardless of riboflavin treatment. We evaluated the impact of topical collagenase on eyes treated with KeraVio without riboflavin drops, but the corneal biometry needs to be clarified.

This study aimed to evaluate corneal biometry after treatment with both collagenase and VL irradiation in rabbit eyes.

## Methods

Nine female Japanese White rabbits weighing 1.5–2.0 kg were used in the study. All animals were healthy and free of ocular disease. In six rabbits, the right eyes were treated with collagenase type II, and the left eyes were treated as the control group (*n* = 6 in each group). In the other three rabbits, KeraVio treatment was applied to both eyes after topical collagenase application (*n* = 6). All animals were treated according to the Association for Research in Vision and Ophthalmology Statement for the Use of Animals in Ophthalmic and Vision Research. This study was approved by the Review Board at Life Science Laboratories, Ltd: #18-20.

A topical anesthetic consisting of 0.4% oxybuprocaine hydrochloride was applied to the eyes. After epithelial debridement, corneal trephines were placed on the corneas. In the collagenase group, a 10 mg/ml collagenase type II solution (Worthington, Lakewood, NJ) in balanced salt solution and 15% dextran was applied to the surface of the corneas for 30 min with corneal trephines, as reported by Hong et al. (5). We chose the epithelium removal technique to encourage collagenase to penetrate corneal stroma, in terms of drug delivery. The solution was then removed with cotton swabs, and the corneas were rinsed with 0.9% sodium chloride solution. The control eyes were subjected to the same protocol but without collagenase type II in the applied solution. The KeraVio process began with the application of collagenase in the same manner as in the collagenase group. VL irradiation (375 nm) was applied using a single VL diode (Nitride Semiconductors Co., Ltd., Tokushima, Japan), with an irradiance of 0.31 mW/cm^2^ for 180 min at a distance of 60 cm from the cornea (total energy dose, 3.3 J/cm^2^). KeraVio treatment using only VL irradiation was continued for 7 days (total energy dose, 23.4 J/cm^2^) (9). The experiments were carried out at room temperature (20 ± 1°C).

Before surgery, the rabbit eyes were subjected to slit-lamp examinations to identify the evidence of conjunctival injection, corneal infiltration, and corneal stromal inflammation. These examinations were repeated every day during the 7-day study to assess ocular safety; an examination was carried out before the collagenase treatment and repeated every day during the 7-day study. From day 1 to day 6, measurements of corneal parameters were not performed, but slit-lamp examinations were carried out daily. Corneal keratometry and the central corneal thickness (CCT) were recorded at the baseline (the day before treatment) and 7 days after treatment using a handheld keratometer (Retinomax 3; Righton, Tokyo, Japan) and an ultrasound pachymeter (SP-100; Tomey, Nagoya, Japan), respectively, under topical anesthesia. Although the maximum measurable refractive astigmatism of the Retinomax 3 was up to 12 dioptres (D), we modified its limit and reset the maximum value up to 30 D. Regarding the axis orientation of corneal astigmatism, no data were provided because of unreliable alignment using a handheld keratometer. Three measurements were taken at each time point, and the mean value of each parameter was recorded along with the change in its value. The mean steep keratometry (Ks), corneal astigmatism, CCT, and changes in each parameter were evaluated. The axial length was also recorded at day 7 after treatment using a digital caliper. In this study, the axial length was defined as the distance from the corneal apex to the scleral apex near the optic nerve.

The rabbits were euthanized with an intravenous overdose of sodium pentobarbital on day 7. The corneas were harvested en bloc along the sclera. A 2–3 mm scleral rim was preserved, and the cornea was attached along a custom-made scale. Then, a 5-mm-wide corneal strip was resected vertically along the cornea. The corneal strips were clamped vertically at a distance of 5 mm between the jaws. The CCT at day 7 in each cornea was used to calculate the cross-sectional area of the corneal strip. After the prepared corneal strip was placed on a computer-controlled electronic universal testing machine (TA, XTplusC Texture Analyser^TM^, Stable Micro Systems, Ltd., London, UK) on day 8, a fixture was applied to hold the corneoscleral limbus of the corneal strip during a uniaxial tensile test. For the actual measurement, the sample was stretched at a velocity of 1.8 mm/min up to a maximum force of 5 N. The elastic modulus was defined as the ratio of tensile stress (amount of force causing deformation per unit trans-sectional area of the corneal strip) to tensile strain (percentage change in the length caused by the stress). For the subsequent statistical analysis, the elastic modulus was consistently evaluated at 3, 5, and 10% strain.

Analyses were performed with Statistical Analysis Software (version 9.4; SAS Institute, Cary, NC). The outcome measures were reported as the mean ± standard deviation. The normality of all data samples was first checked by the Kolmogorov–Smirnov test. The Mann–Whitney U-test was used to compare the data between the two groups. A *P*-value of < 0.05 was considered statistically significant. The sample size (*n* = 6 in each group) in this study offered 88% statistical power at the 5% level to detect a 12-D difference in corneal astigmatism between the two groups when the standard deviation of the mean difference was 6 D. To determine the repeatability of Ks and astigmatism, intraclass correlation coefficients (ICCs) were calculated for the three repeated measurements at the baseline point in each eye. The ICC was determined based on the analysis of variance for mixed models for each situation, as proposed by Bartko and Carpenter (13). The ICC ranges from 0 to 1 and is commonly classified as follows: an ICC < 0.75 indicates poor repeatability; an ICC of 0.75 to < 0.90 indicates moderate repeatability; and an ICC >0.90 indicates high repeatability.

## Results

After collagenase treatment, daily slit-lamp examinations showed no conjunctival infection, corneal infiltration, or corneal stromal inflammation throughout the follow-up period. Fluorescein staining examinations showed complete corneal epithelial healing at ~7 days after the surgery.

At the baseline, the Ks values in the collagenase and KeraVio with collagenase groups did not differ from that in the control group (*P* = 0.058 and *P* = 0.180, respectively). Table 1 shows the Ks values from the baseline throughout the 1-week observation period. The change in Ks in the collagenase and KeraVio with collagenase groups was significant on day 7 compared with that in the control group (each *P* = 0.002).

**Table 1:** Changes in steep keratometry over time.

	**Baseline dioptres (D)**	**1 week (D)**	**Change from baseline to 1 week (D)**	^*^P-value	**^†^*P-*value**
Collagenase	54.73 ± 0.74	61.81 ± 5.51	7.08 ± 5.58	0.002	n/a
KeraVio with collagenase	56.49 ± 3.77	62.59 ± 6.29	6.10 ± 5.83	0.002	0.699
Control	57.62 ± 2.01	53.06 ± 1.21	−4.56 ± 2.14	n/a	n/a

* Compared with the control group.

† Comparison between collagenase with and without KeraVio.

D, diopters; n/a, not applicable.

At the baseline, corneal astigmatism in the collagenase and KeraVio with collagenase groups did not differ from that in the control group (*P* = 0.065 and *P* = 0.197, respectively). No significant difference was found in baseline corneal astigmatism between the KeraVio and collagenase groups (*p* = 0.055). Table 2 demonstrates the corneal astigmatism values from the baseline throughout the 1-week observation period. The changes in corneal astigmatism in the collagenase and KeraVio with collagenase groups were significant on day 7 compared with that in the control group (each *P* = 0.002).

**Table 2:** Changes in corneal astigmatism over time.

	**Baseline (D)**	**1 week (D)**	**Change from baseline to 1 week (D)**	^*^P-value	**^†^*P-*value**
Collagenase	1.74 ± 0.43	20.13 ± 7.37	18.39 ± 7.51	0.002	n/a
KeraVio with collagenase	5.63 ± 1.55	20.50 ± 5.73	14.87 ± 5.19	0.002	0.240
Control	4.04 ± 1.82	2.94 ± 0.82	−1.11 ± 2.50	n/a	n/a

* Compared with the control group.

† Comparison between collagenase with and without KeraVio.

D, diopters; n/a, not applicable.

Table 3 shows the repeatability of Ks and corneal astigmatism with the handheld keratometer. The repeatability of these parameters was excellent, with an ICC higher than 0.900.

**Table 3:** Repeatability of three measurements at the baseline by the ICC.

	**ICC (95% CI)**
Steep keratometry	0.908 (0.872–0.937)
Corneal astigmatism	0.904 (0.883–0.929)

ICC, intraclass correlation coefficient; CI, confidence interval.

At the baseline, the CCT in the collagenase and KeraVio with collagenase groups did not differ from that in the control group (*P* = 0.150 and *P* = 0.355, respectively). Table 4 presents the CCT values from the baseline throughout the 1-week observation period. The change in the CCT in the collagenase group was significantly different on day 7 compared with that in the control group (*P* = 0.002) but not the KeraVio with the collagenase group (*P* = 0.132).

**Table 4:** Changes in central corneal thickness over time.

	**Baseline (μm)**	**1 week (μm)**	**Change from baseline to 1 week (μm)**	^*^P-value	**^†^*P-*value**
Collagenase	380.33 ± 31.13	289.67 ± 46.99	−90.67 ± 57.11	0.002	n/a
KeraVio with collagenase	377.00 ± 27.88	290.00 ± 81.69	−87.00 ±101.48	0.132	0.818
Control	341.67 ± 20.43	341.00 ± 14.76	−0.67 ± 17.10	n/a	n/a

* Compared with the control group.

† Comparison between collagenase with and without KeraVio.

n/a, not applicable.

Table 5 shows the elastic modulus of the treated corneas at 3, 5, and 10% strain. We found significant differences in stress values between the collagenase and control groups at 3, 5, and 10% strain (*P* = 0.004, 0.004, and 0.002, respectively).

**Table 5:** Comparison of the elastic modulus in each group.

	**3% (kPa)**	^*^P-value	**^†^*P-*value**	**5% (kPa)**	^*^P-value	**^†^*P-*value**	**10% (kPa)**	^*^P-value	**^†^*P-*value**
Collagenase	96.60 ± 51.87	0.004	n/a	104.01 ± 58.28	0.004	n/a	121.48 ± 81.08	0.002	n/a
KeraVio with collagenase	262.84 ± 170.17	0.394	0.065	289.69 ± 213.20	0.310	0.132	466.35 ± 384.02	0.310	0.132
Control	354.40 ± 190.22	n/a	n/a	407.89 ± 220.83	n/a	n/a	604.48 ± 280.05	n/a	n/a

* Compared with the control group.

† Comparison between collagenase with and without KeraVio.

n/a, not applicable.

**Figure 1:**
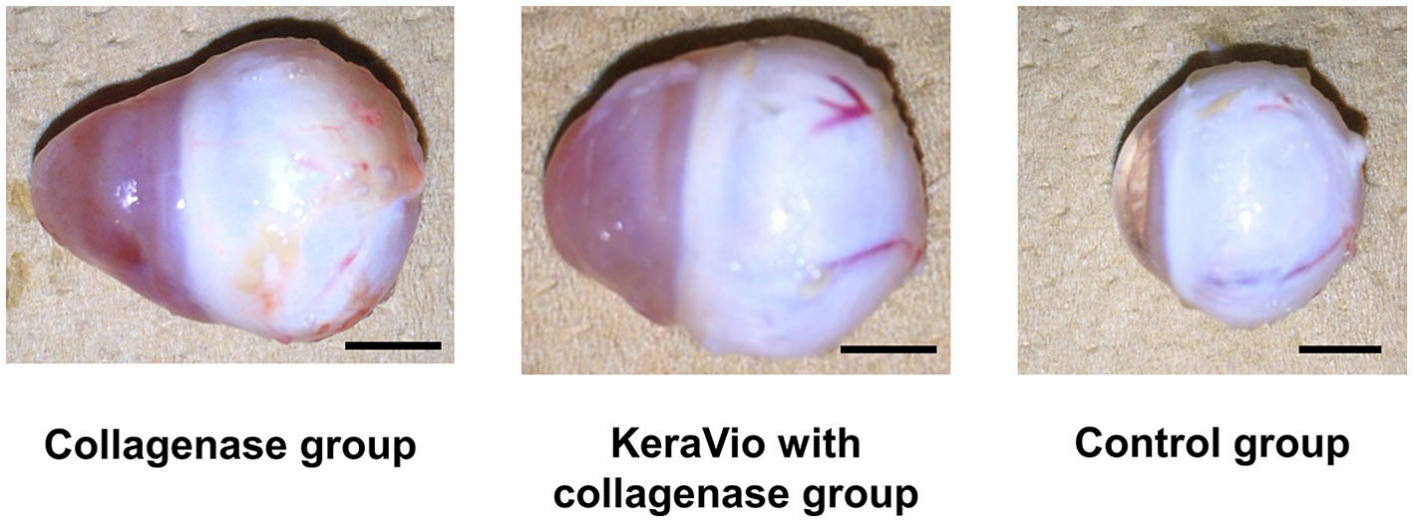
Whole eyes demonstrating the axial length in the collagenase, KeraVio with collagenase, and control groups. The scale bar represents 5 mm.

Table 6 presents the average axial length at day 7. The axial length in the collagenase and KeraVio with the collagenase groups was significantly longer than that in the control group (*P* = 0.004 and 0.009, respectively). Figure 1 shows a representative whole collagenase-treated eye, KeraVio–collagenase-treated eye, and control eye after euthanasia.

**Table 6:** Average axial length at day 7 in each group.

	**Axial length at day 7 (mm)**	^*^P-value	**^†^*P-*value**
Collagenase	20.28 ± 1.63	0.004	n/a
KeraVio with collagenase	19.05 ± 1.51	0.009	0.251
Control	16.45 ± 1.37	n/a	n/a

* Compared with the control group.

† Comparison between collagenase with and without KeraVio.

n/a, not applicable.

When we compared each parameter between the collagenase and KeraVio with collagenase groups, there was no significant difference in the change in Ks, corneal astigmatism, CCT, and corneal stiffness at 3, 5, or 10% strain, or axial length.

## Discussion

In the present study, collagenase treatment induced a significant increase in Ks (7.08 D) at day 7, which is consistent with the clinical characteristics of irregular corneal topography. Hong CW et al. (5) reported that collagenase treatment resulted in a significant increase in corneal curvature in human corneas by 6.6 ± 1.1 D. Wang X et al. (7) demonstrated that rabbit corneas exposed to collagenase *ex vivo* showed a significant decrease in CCT. Regarding corneal thickness during accelerated CXL in clinical KC, Ocak et al. (10) found a significant decrease at the end of ultraviolet-A irradiation. They also reported a significant decrease in endothelial cell density postoperatively (11). However, we have no data on corneal thickness and endothelial cell density before and after CXL treatments. Some studies have also focused on the relationship between corneal keratometry and time (5, 6). Our study also revealed a similar decrease in Ks in the control group. Although previous studies evaluated keratometry and corneal thickness after collagenase treatments, corneal astigmatism was not clarified (5, 6). To imitate KC, we measured corneal astigmatism as a lower-order aberration. Higher-order aberrations were not evaluated in this study because corneal topography was not available. Compared to the control group, the collagenase group exhibited a significant increase in corneal astigmatism (18.39 D) at day 7.

Regarding axial length, we previously reported that VL irradiation may have the potential to retard myopia progression (14). We assessed the impact of VL irradiation on the change in axial length in eyes treated with collagenase. However, there was no significant difference in the axial length at day 7 between eyes treated with collagenase with and without KeraVio. Although we should have assessed various collagenase concentrations and exposure times in this study, we used the same methods as those of previous investigations (5, 6). The change in the axial length was a secondary outcome after collagenase administration. Unfortunately, we did not use the ultrasound method to measure the axial length in this study. Instead, we are conducting a subsequent investigation to evaluate scleral and corneal morphological changes after collagenase treatment using ultrasound.

We found significant differences in the elastic modulus between the collagenase and control groups at 3, 5, and 10% strain. Generally, when the elastic modulus of corneal strips is compared, the value at 10% strain is useful and is commonly recorded (15, 16). In our study, the stress–strain curves showed an exponential increase, as in the previous investigation by Wollensak et al. (15). Compared to the results in a relevant report by Qiao et al. (6), the elastic modulus in our study demonstrated high standard deviations in each group. The discrepancy might be attributable to differences in the measurement conditions of the stress–strain test. Our higher standard deviations might be related to a day of delay in the measurement schedule due to logistics. Corneal epithelial removal induced discomfort, such as ocular pain in the rabbits, but these effects were controlled using oxybuprocaine drops.

Regarding the impact of KeraVio with collagenase on the cornea, no significant difference was found in any parameter between the collagenase and KeraVio with collagenase groups. However, the KeraVio with the collagenase group showed a slightly higher elastic modulus at 10% strain than the collagenase group. We suggest that this effect may have been due to the higher concentration of collagenase, a short-term follow-up, and an absence of riboflavin drops in the KeraVio with the collagenase group. The short-term follow-up of 7 days might be a limitation of this study because remodeling in the cornea is unstable. However, the KeraVio treatment provided sufficient VL irradiation for 7 days to produce a total energy dose that was 4.3-fold higher than the dose delivered by the standard Dresden protocol for CXL (23.4 J/cm^2^ vs. 5.4 J/cm^2^). Because the current study did not include corneal changes after the CXL technique, it was difficult to determine whether KeraVio outperformed CXL in terms of preventing corneal changes. We previously compared the elastic modulus after KeraVio and standard CXL treatments without collagenase application using porcine corneas (12). The model of the previous *ex vivo* study (12) differed in terms of species from the rabbit model in this study. Further research is needed to measure the concentration of riboflavin in rabbit corneas *in vivo*. In our previous study without collagenase treatment, the KeraVio without riboflavin (VL irradiation only) and standard CXL groups showed a significantly higher elastic modulus than the control group consisting of normal eyes, whereas no significant difference between the KeraVio and CXL groups was found (12). The preliminary data indicated similar outcomes between the two treatment groups (12). To the best of our knowledge, this is the first study to investigate corneal biometry and biomechanics after treatment with collagenase and with and without KeraVio. We are optimizing the dose of VL irradiation in preclinical investigations to move to clinical studies of KeraVio, which is similar to but distinct from CXL. To evaluate the preliminary efficacy of KeraVio using only VL irradiation without riboflavin drops, we established a collagenase animal model in the rabbit cornea.

The main limitation of the current study is that it did not assess the long-term impact of this novel corneal ectasia treatment. We avoided ethical issues regarding rabbit discomfort. No corneal complications or adverse events were observed during the 7-day follow-up period. This 1-week observation period may be too short to obtain a comprehensive understanding of this enzymatic degradation model, although, in the present study, a significant increase in Ks and a significant decrease in the elastic modulus were observed in 1 week. In addition, long-term observation is necessary for further identification of changes in Ks, corneal astigmatism, and tensile strength, which could confirm the sustainability of this novel model. Additional studies with long-term follow-up periods are warranted to better investigate the reliability and efficacy of this *in vivo* animal model of corneal ectasia. Another minor limitation is that we have no data on histopathological findings in this study. However, previous research demonstrated histopathological findings of loosely arranged collagen fibers and interlamellar clefts after collagenase treatment (6). In this study, we focused on the impact of VL irradiation under collagenase treatment. Further studies should include comprehensive assessments of histopathological images, corneal strips, and results of computer-controlled electronic universal testing machine trials. Another limitation is that in the collagenase treatment-only group, the right eye was treated while the other eye was used as a control, but in the KeraVio group, both eyes were treated when there should have been a similar monocular control.

In conclusion, a method for generating keratometric features of KC was demonstrated in rabbit eyes treated with collagenase. There was no significant difference in the observed elastic behavior of normal and ectatic corneas under physiologically relevant stress levels. KeraVio, including VL irradiation, did not cause regression of corneal steepening in this model. Further study is required to evaluate the efficacy of novel treatments for this disorder.

## Data availability statement

The raw data supporting the conclusions of this article will be made available by the authors, without undue reservation.

## Ethics statement

All animals were treated according to the Association for Research in Vision and Ophthalmology Statement for the Use of Animals in Ophthalmic and Vision Research. This study was approved by the Review Board at Life Science Laboratories, Ltd: #18-20.

## Author contributions

HK, TY, and KT were involved in the design and conducted the study, collection, management, analysis, interpretation of data, preparation, review, and final approval of the manuscript. All authors contributed to the article and approved the submitted version.
